# Open-source software for mouse-tracking in Qualtrics to measure category competition

**DOI:** 10.3758/s13428-019-01258-6

**Published:** 2019-06-13

**Authors:** Maya B. Mathur, David B. Reichling

**Affiliations:** 1 Department of Epidemiology, Harvard T. H. Chan School of Public Health, Boston, MA USA; 2 Quantitative Sciences Unit, Stanford University, 1070 Arastradero Road, Palo Alto, CA 94305 USA; 3 Oral & Maxillofacial Surgery (retired), University of California at San Francisco, San Francisco, CA USA

**Keywords:** Experimental design, Mouse-tracking, Response dynamics, Cognition, Qualtrics, R

## Abstract

Mouse-tracking is a sophisticated tool for measuring rapid, dynamic cognitive processes in real time, particularly in experiments investigating competition between perceptual or cognitive categories. We provide user-friendly, open-source software (https://osf.io/st2ef/) for designing and analyzing such experiments online using the Qualtrics survey platform. The software consists of a Qualtrics template with embedded JavaScript and CSS along with R code to clean, parse, and analyze the data. No special programming skills are required to use this software. As we discuss, this software could be readily modified for use with other online survey platforms that allow the addition of custom JavaScript. We empirically validate the provided software by benchmarking its performance on previously tested stimuli (android robot faces) in a category-competition experiment with realistic crowdsourced data collection.

## Introduction

**Fig. 1:**
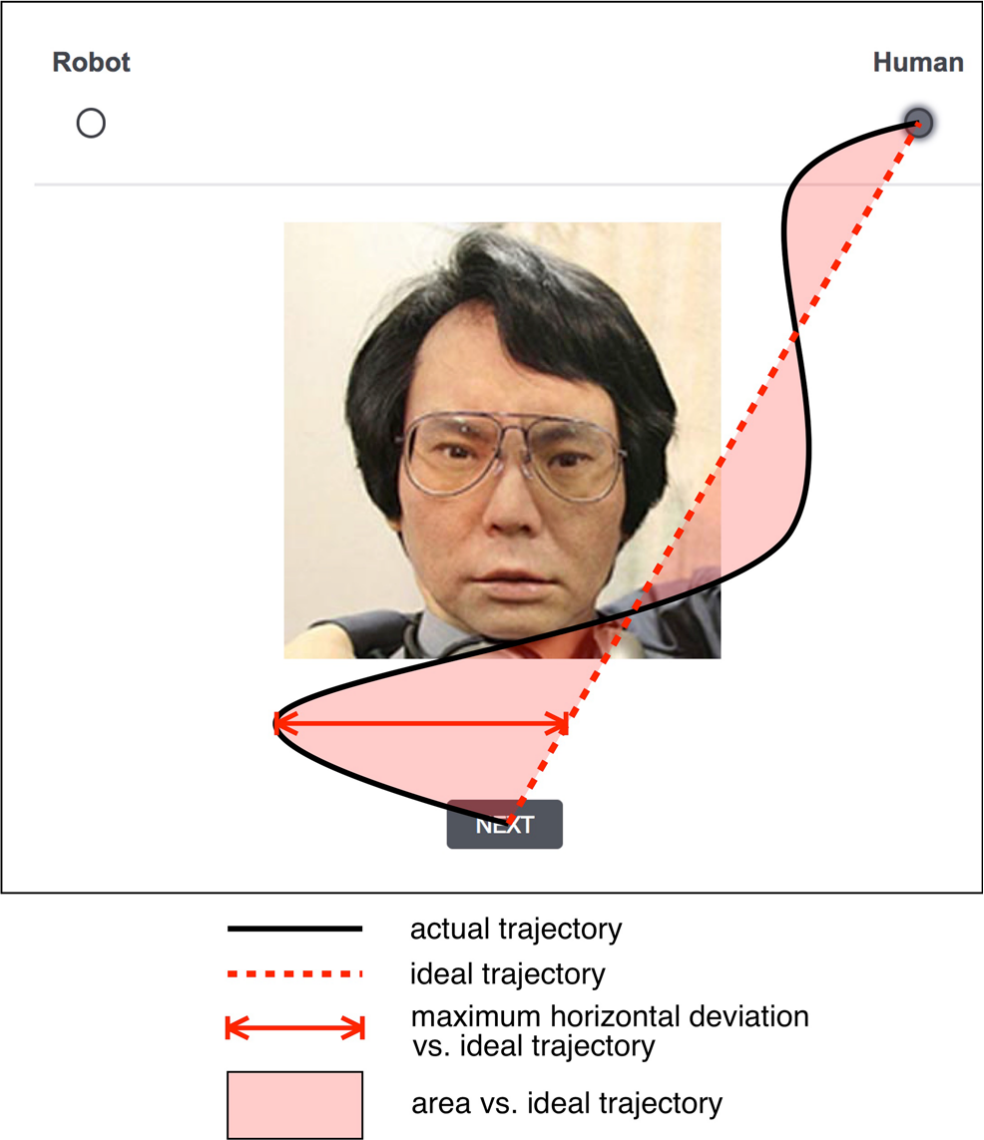
Typical outcome measures for category-competition experiments. In this example, a hypothetical subject’s cursor trajectory suggests initial attraction to the “robot” category, but in an early change of direction, the subject appears to become more attracted to the “human” category. There is a final, weak attraction once again to the “robot” category, but the subject ultimately categorizes the face as “human”. In our implementation, there is a 570-px horizontal distance between the category buttons and a 472-px vertical distance between the category buttons and the middle of the Next button

Capturing rapid, dynamic cognitive processes that may lie outside subjective awareness is a key methodological task in several realms of experimental psychology. One promising method for gaining insight into these processes is to analyze the trajectories of subjects’ mouse cursors as they complete experimental tasks (Freeman & Johnson, 2016). For example, in tasks in which subjects must rapidly categorize stimuli (such as faces) into mutually exclusive, binary categories (such as “male” and “female”), the trajectories of subjects’ mouse cursors as they attempt to rapidly select a category button can serve as direct physical manifestations of cognitive competition between the categories (see Fig. 1 for a hypothetical trial). Stimuli that are difficult to categorize because they are intermediate between the two categories or are atypical exemplars of their category, such as gender-atypical faces, tend to produce mouse trajectories that differ markedly from those produced by stimuli falling clearly into one category (Dale, Kehoe, & Spivey, 2007; Freeman, Ambady, Rule, & Johnson, 2008; Freeman, Pauker, & Sanchez, 2016). That is, the trajectories produced when subjects attempt to categorize ambiguous stimuli will tend to reflect the subjects’ “confusion” and simultaneous or alternating attraction to both categories; these trajectories typically show more changes of direction and greater divergence from the most direct possible trajectory from the mouse cursor’s starting and ending positions. For example, in the hypothetical trial depicted in Figure 1, the subject must attempt to categorize as “robot” or “human” a stimulus depicting an extremely human-like android robot. Mouse-tracking has been used to investigate category competition in diverse subdisciplines, including language processing (Dale & Duran 2011; Farmer, Anderson, & Spivey, 2007; Spivey, Grosjean, & Knoblich, 2005), social judgments of white versus black faces (Wojnowicz, Ferguson, Dale, & Spivey, 2009; Yu, Wang, Wang, & Bastin, 2012), and social game theory (Kieslich & Hilbig, 2014).

Collecting reliable mouse trajectories that are comparable across subjects and trials requires precise control over the visual layout and timing of the experiment, as we will describe. Perhaps for this reason, mouse-tracking experiments to date have usually been conducted in person, with subjects physically present in the lab (with some exceptions, e.g., Freeman et al., 2016). Such settings allow for a consistent visual presentation of the experiment through the use of existing mouse-tracking software (Freeman & Ambady, 2010; Kieslich & Henninger, 2017). In contrast, collecting mouse-tracking data online, for example through crowd-sourcing websites, could allow for much larger samples, greater demographic diversity (Gosling, Sandy, John, & Potter, 2010), and the possibility of implementing the same experiment in multiple collaborating labs without special hardware or software requirements. We are not aware of existing open-source software that is suitable for these settings, that can accommodate common experimental features such as presentation of multiple stimuli and randomization, and that ensures a consistent, validated experimental presentation even when subjects complete the study from their home computers or other devices.

The present paper therefore provides open-source software enabling reliable and precise design of mouse-tracking experiments through the widely used software Qualtrics (Provo, UT, last accessed 10-2018), a graphical user interface that is designed for online data collection that interfaces easily with crowd-sourcing websites such as Amazon Mechanical Turk. Our software pipeline consists of: (1) a premade Qualtrics template containing embedded JavaScript and CSS that manages stimulus presentation, trains subjects on the experimental task, and collects mouse trajectory and time data; and (2) R code to clean, parse, and analyze the data. We present a validation study demonstrating consistent data collection even in relatively uncontrolled online settings and demonstrating that these methods show concurrent validity when benchmarked using previously tested stimuli.

## A basic category-competition experiment

In a standard category-competition experiment, the subject views a series of stimuli presented sequentially on separate pages. The subject must categorize each stimulus by clicking on one of two buttons presented on the left and right sides of the window (Fig. 1). Stimuli are typically chosen such that some fall clearly into one of the categories, while others are ambiguous or difficult to categorize. Ambiguous stimuli are thought to activate mental representations of both categories simultaneously, leading to dynamic competition that manifests in real time as unstable mouse dynamics (Freeman & Johnson, 2016). That is, because the subject is continuously or alternately attracted to both categories, the mouse trajectory may contain frequent direction changes and may diverge substantially from a direct path from the start position to the location of the category button ultimately chosen.

Specifically, past literature (e.g., Freeman et al., 2008) has used several outcome measures to operationalize category competition through mouse dynamics. More ambiguous stimuli typically increase the number of times the subject’s mouse changes directions horizontally (*x-flips*). Additionally, compared to unambiguous stimuli, ambiguous stimuli tend to produce trajectories that diverge more from an “ideal trajectory” consisting of a straight line from the subject’s initial cursor position to the finally chosen radio button (Fig. 1, red dashed line). That is, the *maximum horizontal deviation* between the ideal trajectory and the subject’s actual trajectory (Fig. 1, red solid line), as well as the *area* between the ideal and actual trajectories (Fig. 1, pink shading), are typically larger for ambiguous stimuli. Our implementation calculates these measures using trajectories rescaled to unit length in both the *x*- and *y*-dimensions and calculates the area using Riemann integration. Other outcome measures can include the *maximum speed* of the subject’s cursor (ambiguous stimuli tend to produce higher maximum speeds, reflecting abrupt category shifts (Freeman et al., 2016)) and the total reaction time for the trial (ambiguous stimuli tend to produce longer reaction times). We calculate reaction time as the time elapsed between the start of the trial, after the page is fully loaded, to the time the subject clicks on a button to categorize the stimulus. However, both maximum speed and reaction time have limitations and are perhaps best treated as secondary measures (Freeman et al., 2016).

## How to create and analyze an experiment with our software

Our open-source software provides a user-friendly data collection and analysis pipeline for creating such experiments as follows. All questionnaire and code files are available online (https://osf.io/st2ef/), along with a detailed READ-ME file that users are strongly encouraged to read before implementation. First, the user imports into Qualtrics a template questionnaire implementing the validation study presented below. The key feature is two question “blocks” that present the stimuli sequentially, in randomized order, via Qualtrics’ “Loop & Merge” feature; other blocks in the survey, such as one presenting demographic questions, can be added or removed as needed. The image URLs in the Loop & Merge can simply be edited through the Qualtrics interface to replace the default stimuli. The first block of the questionnaire shows instructions (Online Supplement). Then the first Loop & Merge block presents training stimuli to acclimatize the subject to the experiment, including to alert messages designed to optimize subject behavior for mouse-tracking, detailed in “Optimizing subject behavior for mouse-tracking” below. The second Loop & Merge block of experimental stimuli begins data collection by activating mouse-tracking. The underlying JavaScript that activates mouse-tracking^1^ requires no modification except that global variables specifying the number of training stimuli (howManyPracticeImages, defaulting to 6) and real experimental stimuli (howManyRealImages, defaulting to 10) must be changed to match the number of user-supplied stimuli. Additional parameters that the user can optionally change are listed in Table 1. The Qualtrics template also contains (in the “Look and Feel” section accessible through the Qualtrics user interface) a small snippet of CSS that formats the radio buttons.^2^ The Qualtrics questionnaire is then ready to collect data.

**Table 1:** Modifiable JavaScript global variables

Variable	Default	Meaning
howManyPracticeImages	6	The number of practice stimuli (for which no mouse trajectories will
		be recorded)
howManyRealImages	10	The number of experimental stimuli (for which mouse trajectories will
		be recorded)
maxAnswerTime	5000	The maximum time (ms) that can be spent on a trial.
		Trials with longer answer times will receive a “took too long” alert.
maxLatency	700	The maximum time (ms) after trial onset for which subject can
		leave mouse position unchanged.
		Trials with longer latencies will receive a “started too late” alert.

After data collection, the raw Qualtrics dataset in wide format will contain columns with continuous records of the subjects’ mouse coordinates (xPos and yPos), the absolute time (ms since January 1, 1970, 00:00:00 UTC, which is the standard origin time in JavaScript) at which these coordinates were recorded (t), the times at which each trial began (onReadyTime), and the times at which the subject chose a category button (buttonClickTime). These variables are recorded as a single string for each subject with a special character “a” separating the individual recordings, enabling easy parsing in R or another analysis software. That is, onReadyTime and buttonClickTime are sampled once per trial, while xPos, yPos, and t are sampled as a triplet approximately every 16–18 ms.^3^ Additionally, the user’s browser, browser version, operating system, and browser resolution are recorded. Table 2 provides details on these variables, along with additional variables that are collected in the raw Qualtrics data but were not used in the present analyses.

**Table 2:** Codebook of mouse-tracking, timing, and computing system variables in raw Qualtrics data

Variable	Units	Meaning
xPos	px	*x*-coordinate of cursor relative to upper left-hand
		corner of browser window
yPos	px	*y*-coordinate of cursor relative to upper left-hand corner
time	ms since 1970-01-01	Time at which each coordinate pair was measured
	0:00:00 UTC	
onLoadTime	ms since 1970-01-01	Time at which page for each trial started loading
	0:00:00 UTC	
onReadyTime	ms since 1970-01-01	Time at which the page for each trial was loaded
	0:00:00 UTC	(beginning of trial)
buttonClickTime	ms since 1970-01-01	Time at which subject made category decision
	0:00:00 UTC	(end of trial)
pageSubmitTime	ms since 1970-01-01	Time at which subject proceeded to next trial by
	0:00:00 UTC	clicking “Next”
windowWidth	px	Width of subject’s browser window at beginning of trial
windowHeight	px	Height of subject’s browser window at beginning of trial
alerts	N/A	Alerts received during each trial:
		0 = none
		1 = started too early
		2 = started too late
		3 = surpassed time limit for trial
		4 = window too small to fully display experiment
latency	ms	Time between onReadyTime and first mouse move
stimulusOrder	N/A	Stimulus URLs for each trial in the order presented to
		subject
browser_Browser	N/A	Internet browser
browser_Version	N/A	Browser version
browser_Operating.System	N/A	Operating system
browser_Resolution	N/A	Browser resolution

The R code in data_prep.R automatically checks the data for idiosyncratic problems, returning a list of subjects flagged for possible exclusion, along with reasons (see “Special considerations for online use” below for details). The R code then parses the raw data downloaded from Qualtrics, computes the outcome measures described above, and returns the dataset in an analysis-ready format. Specifically, the code first parses the character-separated strings into a list for each subject, each of which contains a list for each experimental stimulus. For example, a particular subject might have the following *x*-coordinate lists for the first three stimuli (prior to rescaling the trajectories to unit length): 
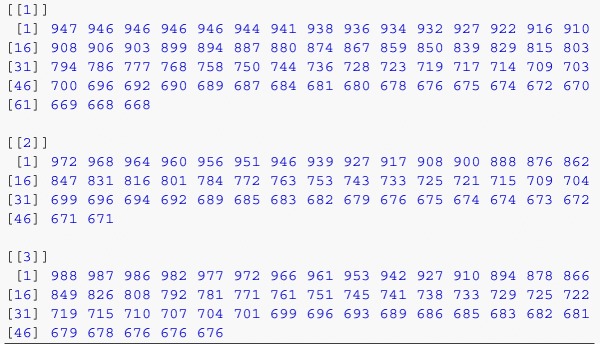


In the process, the code accounts for the possibility of order-randomized Loop & Merge iterates by appropriately reordering the coordinate and time data. The outcome measures are computed for each subject and appended to the wide-format dataset. By default, our analysis code defines the time variable as the time elapsed from the beginning of each trial, specifically the time at which the page was loaded. Note that if the trajectories are to be directly averaged rather than used to compute the outcome measures we describe, the times should be standardized to account for differences in the times elapsed for each trial (Freeman & Ambady, 2010). This can be accomplished simply by passing the argument rescale = TRUE to the function get_subject_lists when parsing the time data. Additional outcome measures, such as trajectory curvature (Dale et al., 2007; Kieslich & Hilbig, 2014; Wojnowicz et al., 2009) or speed profiles throughout a trial (Freeman et al., 2016), could also be easily calculated from the raw coordinate data supplied by the provided R scripts. Finally, the dataset is reshaped into an analysis-friendly long format, such that there is one row for each trial rather than for each subject: 
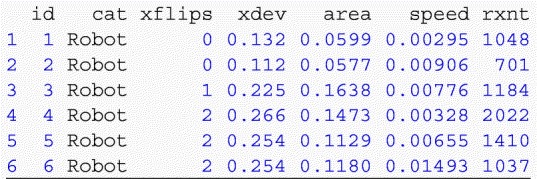


(Note that the outcome measures xflips, xdev, and area are computed using rescaled trajectories, so are unitless.) The code also prints information about alert messages displayed to subjects, discussed in the next section. Although analysis methods will differ by substantive application, we provide an example R file, analysis.R, which conducts the analyses described in “Validation study” below.

## Methodological details

### Optimizing subject behavior for mouse-tracking

If subjects sometimes make their category decisions prior to moving their mouse cursors—that is, if they wait to begin moving their cursors until they have already made a decision—then their mouse trajectories may begin too late to capture dynamic category competition (Freeman et al., 2016). For this reason, at the end of each trial in which the subject took more than 700 ms (by default) to begin moving the cursor, the questionnaire issues a “started too late” alert warning the subject to begin moving the cursor faster at the beginning of each trial. Additionally, to encourage fast decision-making and discourage subjects from taking unscheduled breaks from the experiment, after any trial in which the subject takes longer than 5000 ms (by default) to make a category decision, the questionnaire issues a “took too long” alert reminding the subject to answer more quickly (Freeman et al., 2016). Some investigators choose not to limit total response time (e.g., Kieslich & Hilbig 2014), in which case the parameter *maxLatencyTime* could simply be set to a very large value, such as 50,000 ms. All alerts are recorded in the dataset at the time they are triggered, but to avoid disrupting the subject’s behavior during the trial, they are not displayed onscreen until after the subject selects a category button, but before the subject clicks the Next button to proceed to the next trial. The recorded alert data allow investigators to exclude trials or subjects receiving certain types of alerts if desired. The full text of all alert messages appears in the Online Supplement.

### Special considerations for online use

As mentioned, allowing subjects to complete the experiment on their own devices, rather than in a controlled lab setting, poses several challenges to collecting reliable and precise mouse-tracking data. For example, the software cannot precisely position the subject’s cursor at the start of each trial; browsers do not provide this functionality to preclude malicious misuse. Furthermore, the experiment interface is displayed with the same pixel dimensions for every subject and trial, regardless of the size and resolution of each subject’s screen, potentially yielding interfaces of somewhat differing visual sizes for different subjects. Fixing the visual size, rather than the pixel dimensions, of the experiment interface across subjects was not feasible because the survey software does not have reliable access to data on each subject’s screen size and resolution. Additionally, if subjects attempted to complete the experiment with a browser window that is smaller than the size of the experiment interface (for example, because their devices’ screens are physically too small), then they might have to scroll in the middle of each trial, leading to non-continuous mouse trajectories and erroneous reaction times.

Our JavaScript implementation addresses each of these possibilities. To ensure that the cursor starts in an approximately fixed location, the Next button, which is the necessary ending point for the cursor on every trial, is positioned in the same location on every trial. Furthermore, if the subject moves the cursor away from this position before the next trial begins (i.e., while the page is loading), the questionnaire issues a “started too early” alert to warn the subject not to begin moving the cursor before the page is loaded. During the first training trial, the code checks the pixel dimensions of the subject’s browser window, and if the window is smaller than the expected pixel dimensions of the experiment interface, the questionnaire issues an alert instructing the subject to increase the window size until the stimulus image, both radio buttons, and the submit button are fully visible. On subsequent trials, the subject’s ability to scroll is disabled, such that subjects using devices with too-small screens or browser windows will not have access to the Next button and will thus be unable to proceed through the experiment.

As mentioned above, the use of fixed pixel dimensions does not guarantee that the visual distance between the buttons will be the same for every subject due to the many possible combinations of different physical dimensions of computer monitors and different pixel-per-inch resolutions. In addition, some subjects might use their browser’s zoom function, changing both the pixel distances and the visual distances. Therefore, our R analysis code by default rescales all trajectories to unit length in both the *x*- and *y*-dimensions. However, the validation study described in “Validation study” below found systematically larger values of the outcome measures for subjects with trajectories suggesting non-standard pixel scaling due, for example, to zooming typically showed larger values of the outcome measures. These differences persisted despite that the trajectories had been rescaled to unit length. Importantly, despite these mean differences on the outcome measures, the key stimulus ambiguity effects were comparable between subjects with non-standard pixel scaling and subjects with standard pixel scaling. In practice, then, investigators might choose to simply adjust analysis models for covariates indicating whether a subject had non-standard pixel scaling (operationalized as having unexpectedly large or small pixel distances between the starting and ending *x*-coordinates on any trial) and whether a subject had ever had a too-small window; this is the approach we adopt in the validation study. Because the experimental manipulation is randomized, these idiosyncrasies of the visual display size effectively introduce “non-differential” noise in the continuous outcome measures, in which case the estimate for the effect of stimulus ambiguity remains unbiased even without adjustment for the scaling and window size variables (Rothman, Greenland, Lash, & et al. 2008). Thus, estimates should be comparable across samples with different frequencies of non-standard scaling and too-small windows. However, adjusting for these variables as “precision covariates” may improve statistical power by removing some of the residual variation on the outcome measures that is due to these visual idiosyncrasies rather than to stimulus ambiguity. The provided R code automatically includes these two indicator variables (called weird.scaling and wts, respectively) in the prepared long-format dataset. Alternatively, subjects displaying these idiosyncrasies could simply be excluded.

As an additional data quality concern in online settings, it is sometimes possible for automated “bots” to complete Mechanical Turk tasks, yielding invalid data (Difallah, Demartini, & Cudré-Mauroux, 2012). Because bots do not physically use computer mice or trackpads to proceed through the questionnaire, but rather select buttons directly, they would not provide any mouse trajectories at all for our data collection system to erroneously record. If a bot managed to complete the questionnaire and respond to any alerts in the process, our data preparation script would automatically flag its data for exclusion due to missing trajectories.

### Extensions to other survey platforms

This software is tailored to the Qualtrics survey platform. However, because the specialized functions that manage the collection of mouse trajectory and timing data are entirely contained in the JavaScript, this code could be readily adapted to other online survey platforms or custom experimental interfaces as long as they are able to: (1) support addition of custom JavaScript, and provide a JavaScript API with basic functions similar to Qualtrics’ addOnReady, addOnLoad, disableNextButton, enableNextButton, and setEmbeddedData; (2) present multiple stimuli iteratively, while recording their possibly randomized order; and (3) display the experiment at fixed pixel dimensions. In short, to use this software on another platform, an investigator would need to use that platform’s user interface to adjust the questionnaire display and flow to imitate our Qualtrics-implemented design and would need to add our custom JavaScript, replacing the small number of calls to the Qualtrics API with the relevant functions for the investigator’s own platform. Additionally, the values of some JavaScript global variables related to the display of the experiment, such as minWindowWidth and minWindowHeight, might require modification. The JavaScript is thoroughly commented to facilitate such adaptation and further modification by other users. Finally, it would also be possible for investigators with experience coding in HTML to create a simple survey platform, incorporating our Javacsript code, that could be hosted on their own servers or used to run subjects in the lab.

### Limitations

Our implementation has limitations. Occasional idiosyncrasies (e.g., extremely poor quality connections, use of proxy servers) can cause losses of coordinate data for some trials or subjects. Our R code automatically checks for subjects with these data losses and creates a list of subject IDs that should be excluded, along with reasons for exclusion. The validation study presented below suggested that these issues affect a small fraction of trials for approximately 10% of subjects when data are collected in an uncontrolled crowdsourcing setting. A conservative analysis approach, which we adopt in the validation study, could be to exclude every subject with data losses on any trial.

Additionally, our implementation cannot control subjects’ individual mouse speed settings. That is, different mice and trackpads may be set to respond with larger or smaller onscreen movements for any given physical movement of the subject’s hand, and these differences in mouse dynamics could affect the confusion measures. Because our implementation collects data through an Internet browser, it is not able to measure subjects’ mouse speeds independently of, for example, their hand speeds. However, like the visual idiosyncrasies produced by non-standard pixel scaling or small browser windows, we would expect differences in mouse speed settings to introduce only non-differential noise in the outcomes and thus not compromise estimation of stimulus ambiguity effects (albeit with some loss of statistical power).

Last, although our implementation appears to perform reliably across common browsers (see “Effect of stimulus ambiguity on mouse trajectories”), it is incompatible with Internet Explorer; subjects running Internet Explorer will be unable to proceed through the questionnaire, and no data will be collected. (At present, Internet Explorer has only a 3% share of browser usage worldwide (“Browser market share worldwide”, 2018). Finally, subjects with very slow Internet connections, causing image stimuli to load slowly, may receive a large number of “started too late” alerts, although their data will otherwise be useable. In practice, subjects with a high frequency of “started too late” alerts could be discarded if this were of concern.

## Validation study

### Design and subjects

**Fig. 2:**
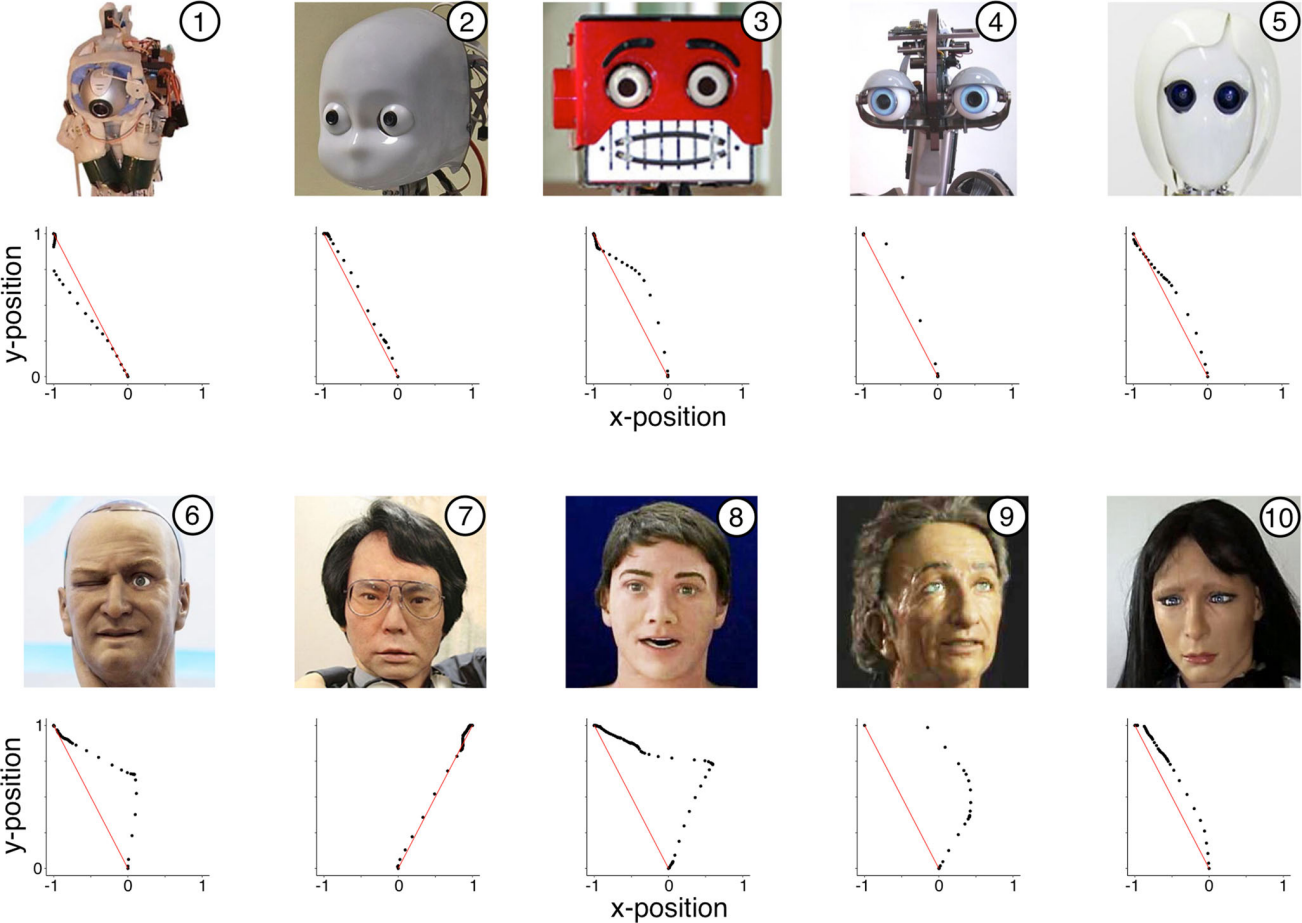
Mouse trajectories for a single subject categorizing unambiguous (*top row*) versus ambiguous (*bottom row*) humanoid robot faces. Trajectories have been rescaled to unit length in both the *x*- and *y*-dimensions

To validate the provided software, we used it to perform a simple category confusion experiment using image stimuli depicting the faces of humanoid robots ranging from very mechanical to very humanlike. Previous work (e.g., Mathur & Reichling 2016; Mathur & Reichling 2009) suggests that humanoid robot faces that closely, but imperfectly, resemble humans—those occupying the “Uncanny Valley” (Mori, 1970)—can provoke intense feelings of eeriness, dislike, and distrust in human viewers. One mechanism of these negative reactions may be that robots occupying the Uncanny Valley provoke category confusion, which may itself be aversive (Yamada, Kawabe, & Ihaya, 2013). In partial support for this hypothesis, Mathur and Reichling (2016) found that robot faces in the Uncanny Valley elicited the most category confusion. As a validation, we attempted to conceptually reproduce Mathur and Reichling (2016)’s findings using the mouse-tracking software presented here. From Mathur and Reichling (2016)’s stimuli, we arbitrarily selected five “unambiguous” faces not occupying the Uncanny Valley (Fig. 2, row 1) and five “ambiguous” faces occupying the Uncanny Valley (Fig. 2, row 2). Given previous findings regarding these faces (Mathur & Reichling, 2016), we expected mouse trajectories to indicate greater average confusion for ambiguous faces vs. unambiguous faces. We analyzed mouse trajectories from *n* = 188 United States subjects recruited on Amazon Mechanical Turk from among users with a prior task approval rating of at least 95%. We compensated subjects $0.25 to complete the study and set a time limit of 20 minutes for the entire task to discourage subjects from taking long breaks from the study. Subjects used the template Qualtrics questionnaire provided here to categorize each face as either a “robot” or a “human”. We randomized the order of stimulus presentation for each subject. A link to a live demonstration version of the questionnaire is provided at https://osf.io/st2ef/.

### Statistical analysis

We regressed each of the five outcome measures described in “A basic category-competition experiment” on a binary indicator for stimulus ambiguity. For ease of interpretation, we first standardized the four continuous outcome variables (area, maximum x-deviation, peak speed, and reaction time); thus, their coefficients represent the average number of standard deviations by which the outcome measure was larger for ambiguous versus unambiguous trials. Regression models were semiparametric generalized estimating equations (GEE) models with a working exchangeable correlation structure and robust inference, and the unit of analysis was trials (1880 observations). We chose the GEE specification in order to account for arbitrary correlation structures within subjects and within stimuli, as well as to avoid making distributional assumptions on the residuals for highly skewed outcomes such as reaction time. Models for continuous outcomes used the identity link, while the model for *x*-flips used the Poisson link. To account for residual variation in the visual display size of the experiment as described in “Special considerations for online use” above, each outcome model included main effects of indicator variables for non-standard pixel dimensions and for too-small browser windows (the variables weird.scaling and wts), as well as all possible interactions among these nuisance variables and the stimulus ambiguity indicator. As a sensitivity analysis, we also performed the analyses excluding all such subjects (for an analyzed *n* = 103) rather than adjusting for the nuisance covariates, yielding nearly identical point estimates and inference.

### Results

#### Descriptive measures

Table 3 displays demographic characteristics of the analyzed subjects, as well as their Internet browsers and operating systems. We collected data on 203 subjects (using an a priori sample size determination of *n* = 200) and excluded 24 due to idiosyncratic timing issues, yielding an analyzed sample size of 188. These exclusion criteria are conservative in that we excluded all trials for any subject with these problems on any trial, even if only a small number of trials were affected. As discussed in “Special considerations for online use”, our questionnaire also collects data on scaling and window size idiosyncrasies that do still allow for normal data collection but that could in principle affect the confusion measures; of the analyzed subjects, 18 had a too-small window on at least one trial, and 77 had non-standard pixel dimensions on at least one trial. No subject’s data indicated a clear violation of the instructions, so we compensated all subjects who completed the study on Amazon Mechanical Turk.

**Table 3:** Demographics and computing system characteristics for subjects in validation study

	Overall
Total N	188
Age (mean (SD))	36.80 (11.73)
Education (n (%))	
Did not graduate high school	2 (1.1)
Graduated 2-year college	35 (18.6)
Graduated 4-year college	75 (39.9)
Graduated high school	54 (28.7)
Post-graduate degree	22 (11.7)
Female (mean (sd))	0.52 (0.50)
Race (n (%))	
Black/African American	16 (8.5)
Caucasian	144 (76.6)
Native American	8 (4.3)
East Asian	12 (6.4)
Hispanic	14 (7.4)
Middle Eastern	4 (2.1)
Southeast Asian	3 (1.6)
South Asian	2 (1.1)
Browser (n (%))	
Chrome	153 (81.4)
Edge	2 (1.1)
Firefox	33 (17.6)
Operating system (n (%))	
Chrome OS	7 (3.7)
Linux	6 (3.2)
Macintosh	19 (10.1)
Windows	155 (82.4)

Across all trials, subjects used a median browser window height of 775 px (25th percentile: 726 px; 75th percentile: 938 px) and a median window width of 1532 px (25th percentile: 1366 px; 75th percentile: 1846 px). Across all trials, the median reaction time was 1170 ms (25th percentile: 859 ms; 75th percentile: 1628 ms). The average latency (that is, the time elapsed between the beginning of the trial and the subject’s first mouse movement) was 442 ms (25th percentile: 87 ms; 75th percentile: 640 ms), which is short enough to suggest that the mouse trajectories would have captured dynamic competition processes occurring almost immediately after stimulus presentation. Across all sampled mouse coordinate pairs, the median sampling rate was 17 times per second (25th percentile: 16 ms; 75th percentile: 18 ms). To provide some reference for the frequency of alert messages that can be expected, 68% of trials received no alerts, and the remaining 32% of trials received a median of 1 alert (of a maximum of 4).^4^ Table 4 displays the relative frequencies of each alert type among all alerts received, and Table 5 displays the percent of subjects receiving each alert type at least once. The fairly high frequency of alerts is to be expected: as discussed in “Optimizing subject behavior for mouse-tracking”, the alerts, particularly those instructing the subject to begin moving the cursor sooner or to avoid moving it before the trial is fully loaded, are designed to optimize subject behavior rather than to indicate invalid data.

**Table 4:** Summary of all 711 alert messages received in validation study across all 1880 trials

Alert type	% of all alerts received
Started too early	40
Started too late	31
Surpassed trial time limit	8
Window too small	21

**Table 5:** Percent of subjects (*n* = 188) receiving each type of alert message at least once across 10 trials

Alert type	% of subjects
Started too early	60
Started too late	58
Surpassed trial time limit	20
Window too small	10

#### Effect of stimulus ambiguity on mouse trajectories

**Fig. 3:**
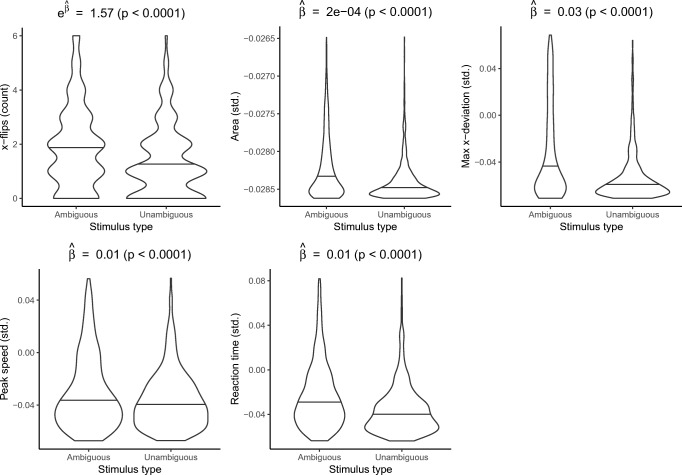
Violin plots showing standardized outcome data for 1880 trials (188 subjects) for ambiguous versus unambiguous face stimuli. *Violin contours* are mirrored kernel density estimates. *Horizontal lines* within violins are medians. $\widehat {\beta }$ = GEE estimate of mean difference (ambiguous - unambiguous); *p* = *p* value for difference estimated by robust GEE inference

As a visual example of the mouse trajectories, Fig. 2 shows unit-scaled trajectories from the fifth subject. For this subject, ambiguous faces 6, 8, and 9 in particular elicited mouse trajectories characteristic of substantial category confusion, evidenced by *x*-flips and large deviations from the ideal trajectory. (The reason for the rightward trajectory for face 7 is that the subject classified this face as “Human”, whereas all the other faces were classified as “Robot”.) Figure 3 aggregates outcome data across subjects in violin plots that display the medians of each standardized outcome measure for ambiguous versus unambiguous stimuli, as well as density estimates of their distributions. These results indicate visually that each measure of confusion was on average higher for ambiguous versus unambiguous stimuli. That is, aligning with the predicted results discussed in “A basic category-competition experiment”, subjects’ cursors appeared to make more horizontal changes of direction, to make less direct paths, and to reach higher peak speeds for ambiguous versus unambiguous stimuli, and furthermore trials for ambiguous stimuli elicited longer reaction times. Point estimates from the GEE models of the mean difference for each confusion measure for ambiguous versus unambiguous stimuli (Fig. 3) were in the predicted direction for all stimuli (with *p* < 0.0001 for all outcomes). Collectively, these results suggest that the software and methods presented here adequately capture confusion when implemented through realistic crowdsourced data collection.

#### Consistency of results across computing systems

As a post hoc secondary analysis to assess the consistency of these stimulus ambiguity effects across browsers and operating systems, we refit the regression models including interaction terms of browser (Firefox vs. Chrome) and of operating system (Macintosh vs. Windows) with stimulus ambiguity. The resulting coefficients thus estimate the differences in the stimulus ambiguity effect on confusion between browsers or between operating systems. We excluded subjects who used other, much less common, browsers and operating systems due to their small sample sizes, yielding 1720 trials in this analysis. Across the five outcome models, the browser interaction coefficients ranged in absolute value from 0.03 to 0.28 with *p* values from 0.32 to 0.54, and the operating system interaction coefficients ranged in absolute value from 0.004 to 0.26 with *p* values from 0.14 to 0.62. While this validation study was not specifically powered to assess for differences in results across browsers and operating system effects, these results suggest that any such effects are likely fairly small.

## Footnotes

1 The JavaScript code is already embedded in the template Qualtrics files, but it is also available as standalone files (https://osf.io/st2ef/).

2 The CSS code is also available as a standalone file (https://osf.io/st2ef/).

3 Specifically, our JavaScript function for recording mouse position (getMousePosition) is triggered by “mousemove” events issued by the browser. Therefore, the frequency of mousemove events determines the minimum time interval for measuring mouse position. The current World Wide Web Consortium standards (“UI events”, 2018) do not specify a frequency at which browsers should issue mouse events, but at the time of writing, the most common browsers use a de facto standard 60-Hz rate (to match the most common display screen refresh rate). Other factors may also contribute to the sampling rate, including mouse DPI (the number of positions reported by the mouse per inch of movement), the system’s USB polling rate (how often the mouse is queried for data), and potential variable lag due to high demand on CPU resources. However, in practice we have found that the effect of these factors must be minimal because our median mouse position interval (17 ms) agrees well with the 60-Hz event reporting interval of 16.7 ms.

4 It may appear counterintuitive that a trial could receive all four alerts, including both “Started too early” and “Started too late”. However, this can occur if the subject moves the cursor outside the Next button before the subsequent trial has fully loaded (“Started too early”) but then, once the subsequent trial is loaded, waits too long to move the cursor again (“Started too late”). To avoid confusing the subject, in this situation, only the “Started too early” alert is actually displayed, though both alerts are recorded in the dataset.
